# Spatial Trends in Mortality Convergence: The Cases of France, Italy, and Spain, 1975–2019

**DOI:** 10.1007/s10680-025-09745-7

**Published:** 2025-08-13

**Authors:** Jacob Martin, Carlo Giovanni Camarda, Timothy Riffe

**Affiliations:** 1 Universidad del País Vasco (UPV/EHU), Leioa, Spain; 2 Institut national d’études démographiques, Aubervilliers, France; 3 Ikerbasque (Basque Foundation for Science), Bilbao, Spain; 4 Max Planck Institute for Demographic Research, Rostock, Germany

**Keywords:** Mortality, Spatial inequalities, Convergence, Life expectancy

## Abstract

Spatial differences in mortality are a significant source of inequality in low-mortality countries and are important for public health, regional planning, and subnational population forecasts. Long-term trends in geographic mortality inequalities remain poorly understood, especially from a comparative perspective. In this study, we examine trends in subnational mortality differences in France, Italy, and Spain from 1975 to 2019. We study whether life expectancy has converged or diverged between geographic areas, assess how the geographic mortality gradient has changed, and document age-specific differences. We find convergence in life expectancy in all three countries, although the path of convergence varies between countries and sexes. We find concerning evidence of diverging mortality at younger ages in all three countries. Our results show that even in similar national contexts, very different subnational trajectories in mortality inequalities can occur.

**Supplementary Information:**

The online version contains supplementary material available at 10.1007/s10680-025-09745-7.

## Introduction

### The Importance of Geographic Inequalities in Mortality

Geographic differences in mortality arise from the different spatial distributions of interacting determinants. Underlying determinants include different exposures to environmental hazards and differing genetic makeup (Frenk et al., 1991), and social, economic, cultural, and political factors. Differing levels of economic development and degree of social stratification, varying social and cultural norms, and differing political systems and public policies all interact to create a geographic distribution of risk factors for mortality (Valkonen, 2001). For example, more economically developed areas will provide a higher material standard of living to their inhabitants, which, all else equal, reduces the risk of death. Regional cultures may have different attitudes toward key risk factors like diet or alcohol and tobacco consumption; these may also be influenced by an area’s mix of economic activity. Finally, heterogeneous political systems produce health systems of varying effectiveness and differing public health policies that mediate the risk factors of health and mortality.

While life expectancy increased globally since the twentieth century, inequalities in the pace of mortality reductions persist between and within countries (Moser et al., 2005). Low-mortality countries exhibit substantial gaps in life expectancy by education (Permanyer et al., 2018; Sasson, 2016), income (Brønnum-Hansen, 2017), and occupational class (van Raalte et al., 2018). Spatial inequalities in mortality in many low-mortality countries remain high, and may be increasing (van Raalte et al., 2020; Seaman et al., 2019). Geography can serve as a proxy for other underlying inequalities and, in some countries, it aligns with the main social, economic, and health gradients. Understanding past trends in regional mortality gradients can shed light on the effectiveness of policies aimed at reducing them, as well as possible paths toward lower levels of inequality. Furthermore, increasing inequalities may be an early warning sign of the development of specific epidemiological conditions that have the potential to spread to the national level. Finally, understanding long-term trends in geographic differences in mortality is important for evaluating subnational population projection methodology. For example, Eurostat assumes long-run convergence in mortality in its subnational population projections (European Comission, 2021).

### Theories of Subnational Mortality Trajectories

Theorizing the decline of mortality at the national level has proven an enduring challenge due to the diversity of mortality experiences over time and between countries (Sudharsanan, Aburto, Riffe, & van Raalte 2022; Vallin & Meslé, 2004). Developing a theory of subnational mortality change presents further difficulties given the added complexity that arises when attempting to understand the trajectories of within-country inequalities. Frenk et al. (1991) proposed a theory of health transition taking into account the heterogeneous composition of national populations, which leads to a superposition of epidemiological conditions and compositional changes within the same country. As mortality declines, the decline happens faster among the more advantaged social strata or geographic areas, creating an “epidemiological polarization” that worsens health inequalities within the country even as national average health conditions are improving.

Vallin and Meslé (2005), building on the work of Frenk et al. (1991), theorized this epidemiological polarization as phases of divergence, embedded in a larger divergence-convergence process that emerges from improvements in health and mortality. More favored populations are able to take advantage of mortality-reducing improvements (improved economic conditions, lifestyle changes, technological innovations, public health policies) earlier, leading to a faster pace of mortality reduction, creating divergences with the less favored populations. However, as improvements diffuse to the less advantaged areas and strata, they too experience reductions in mortality, leading to convergence. Vallin and Meslé (2005) hypothesized that this divergence-convergence cycle has accompanied historical reductions in mortality generally, and that this cycle applies to both between- and within-country differences. In particular, if geographic areas within a country have heterogeneous levels of social and economic development, mortality improvements should occur earliest in the most developed areas, leaving behind other areas and driving divergence. Later, the less advantaged areas should “catch up,” creating convergence.

### Evaluating Demographic Convergence

Assessing convergence in demographic characteristics between populations implies comparing trends indemographic quantities, be they observed counts, rates, or summary indicators such as life expectancy. Developing a method to assess convergence presents two primary difficulties, namely the choice of quantity to compare and the method used to measure convergence in that indicator.

Period life expectancy at birth $e_0$ is the most commonly used indicator in studies of mortality convergence. It represents the average age at death implied in the long run by the mortality rates observed in a period (Preston et al., 2001). This measure is favored because (i) it is independent of the population age structure, and (ii) it reduces a complex mortality age pattern into a single annualized value. However, focusing only on $e_0$ can also obscure important aspects of the mortality schedule in a population, such as variability in age at death. In low-mortality populations in recent years, changes in mortality in older ages have driven improvements in $e_0$ (Aburto et al., 2020), so $e_0$ alone is an inadequate indicator of infant, child, and young-adult mortality. Nonetheless, inequalities at younger ages may be of particular concern when assessing inequalities and convergence between populations. It is possible for there to be overall convergence in life expectancy due to convergence in mortality at older ages while infant simultaneously mortality diverges, for example (Borges, 2018).

We have different indices to assess convergence between populations in life expectancy (or any other indicator). The most commonly applied index, known as “sigma convergence,” occurs when a measure of statistical dispersion (for example, the standard deviation) between subnational values of life expectancy decreases over time, implying that differences between populations have narrowed. Another index, termed “beta convergence” (Barro & Sala-i-Martin, 1992), quantifies whether populations with a lower starting level of life expectancy improve faster than those with a higher starting level, leading them to catch up. Beta convergence and sigma convergence each have two limitations. (i) They do not indicate the direction of convergence, which can be upward, when life expectancy is increasing everywhere, or downward, in which life expectancy stagnates or declines in some populations (Bonnet & d’Albis, 2020). (ii) They do not show the extent of crossovers between populations or, more generally, changes in the position of each population relative to the others. A third index, “gamma convergence,” measures changes in the rankings of populations (Borges, 2018; Boyle & McCarthy, 1997). Sigma, beta, and gamma convergence are not the only available approaches. For example, cluster analysis of multiple indicators has been proposed as a method to identify “convergence clubs” or populations whose mortality profiles change in a similar way over time (Debón et al., 2017; Atance et al., 2024).

### Aims

We seek to analyze the trajectories of subnational inequalities in mortality between 1975 and 2019 in three European countries: France, Italy, and Spain. These are neighbors and peer countries with broadly similar population sizes, levels of economic development, and cultural characteristics. Furthermore, the three countries share relatively similar total population mortality trends over the last decades. However, the countries have differing systems of regional government, and subnational socioeconomic inequalities vary in magnitude and nature between countries. The countries also differ in their organization of health systems: in France, the administration and finances of the health system are very centralized (Or et al., 2023), while in Spain and Italy they are more decentralized (Giulio de Belvis et al., 2022; Bernal-Delgado et al., 2018). Thus, we believe the three countries present a rich opportunity for comparison due to these similarities and differences.

Most studies on subnational convergence in mortality have focused only on quantifying amount of convergence in life expectancy over a short period. Fewer studies have examined changes in the geographic gradient of mortality over time. Furthermore, less research has focused on divergence or convergence in mortality beyond summary measures like life expectancy. Finally, comparative studies of long-term subnational convergence are lacking. We endeavor to comprehensively address these limitations by investigating the following research questions:

1. To what extent is mortality converging or diverging between subnational geographic areas in each country?
2. Are geographic gradients in life expectancy stable or changing?
3. What age-specific patterns in geographic mortality inequalities can be identified?

### Evidence

Geographic mortality patterns in France are relatively well-studied, given the availability of a long time series of detailed mortality data. From the mid-19th century through the 20th century, life expectancy between French departments (subnational territorial units whose composition has remained largely unchanged since the end of the 18th century) largely converged (Vallin & Meslé, 2005; Bonnet & d'Albis, 2020), with inequality between departments declining from its highest levels at the beginning of the 19th century. Furthermore, during the 20th century, some mortality gradients present at the beginning of the century reversed during this phase of convergence: in the 1930 s, the advantage of northern departments shifted to disadvantage, and the department of Paris emerged at an advantage (Bonnet & d’Albis, 2020). However, since the mid-1990 s, inequality between departments has widened (Barbieri, 2013; Bonnet & d’Albis, 2020; Breton et al., 2022).

In Italy, territorial inequalities in health and mortality have been a subject of concern since unification in 1861, with contemporaries noting the abysmal health conditions in the southern provinces of Italy (Sormani, 1881). In the 1880 s, all southern Italian regions and Sicily had life expectancies below the national average (Del Panta & Pozzi, 2011). Between the 1870 s and mid-20th century, death rates converged, and by the 1960 s some southern regions had gained a relative advantage with respect to the national average in terms of life expectancy at birth (Di Comite, 1974). Caselli and Egidi (1979) studied between-province differences in life expectancy in the 1970 s, and found a large southern advantage. By the 1990 s, the advantage of the southern provinces had narrowed, while northern provinces improved (Caselli et al., 2003). At the regional level, Carboni et al. (2024) recently found evidence of convergence starting in 1974 but stopping sometime between the 1990 s and early 2000 s. To our knowledge, no study of provincial convergence from the 1970 s to the 2010 s exists. Also, with the exception of Carboni et al. (2024), previous studies were also limited by their cross-sectional nature or focus on only a few single years of data.

In Spain, differences in life expectancy between autonomous communities (the largest subnational territorial aggregation) increased in the 1970s and decreased in the 1980s and 1990s (Valkonen, 2001). An analysis of data at the provincial level found some convergence in life expectancy between 1980 and 2001 (Montero-Granados et al., 2007). As for Italy, the existing evidence for Spain has been limited by the lack of longer, continuous time series of mortality data, and no comprehensive analysis of provincial mortality from the 1970 s until the late 2010 s has been published, so far as we could find.

Studies of geographic convergence in mortality in other European countries have found evidence of convergence in life expectancy during the late 20th century and early 21st century in the Czech Republic (Kašpar et al., 2017), Germany (van Raalte et al., 2020; Hrzic et al., 2023), and the Netherlands (Janssen et al., 2016). Hrzic et al. (2020) found evidence of convergence in life expectancy and age-standardized death rates across European Union (EU) countries and EU Nomenclature of Territorial Units for Statistics level 2 (NUTS-2) regions between the late 1990s and early 2010s, although this result depended on how convergence was assessed. In Southern, Northern, and Central Europe, life expectancy between NUTS-3 regions converged between 1995 and 2019 (Sauerberg et al., 2024). Geographic disparities in life expectancy in Russia decreased in the early 2000s and 2010s (Shchur et al., 2021), although the level of differences remained high compared to Western European countries.

Outside of Europe, large geographic differences in life expectancy have been found in the United States (Ezzati et al., 2008), and mortality patterns have diverged between states since the late 20th century (Fenelon, 2013; Vierboom et al., 2019). A similar recent divergence has occurred between Canadian provinces, after decades of convergence (Ouellette et al., 2012). Latin America is another region that has historically presented stark regional differences in mortality (Frenk et al., 1991; Prata, 1992), and studies of geographic mortality patterns in Brazil have found convergence in life expectancy between regions between 1980 and 2000, followed by divergence in recent decades (Borges, 2018, 2017; Calazans et al., 2023). In Argentina, geographic disparities in life expectancy decreased between 1980 and 2010 (Grushka, 2014).

## Data and Methods

### Harmonized Mortality Data for France, Italy, and Spain

Our analysis concerns subnational territorial units of France, Italy, and Spain. Each country has a different system of territorial administration, and we opted to conduct our analysis at the NUTS-3 level in each country, specifically: At the department (*département*) level for France, and at the province (*provincia*) level for Italy and Spain.

For France, we use death counts and exposure estimates by single year of age and time period at the department level from the French Human Mortality Database (Bonnet, 2020). These data were processed following the Human Mortality Database (HMD) Method Protocol (Wilmoth et al., 2021). This database gives data only for the departments of metropolitan France, and the two departments of the island of Corsica are grouped together. The Metropolis of Lyon and the department of Rhône are also grouped in this data source as a single department, per the definition before 2015.

For Italy, we obtained death counts by single year of age (0,1,...,100+), sex, and province of residence from the Istituto Nazionale di Statistica (Istat). We obtained births by province of residence of the mother from 1971 onward. Before 1981, resident birth records were not classified by sex, so we used the sex ratio of the *presente* or de-facto births to split the resident or de-jure births by sex (Capocaccia & Caselli, 1990). We also obtained the resident population by age (0,1,...,100+), sex, and province from the 1971 and 1981 censuses, as well as official intercensal estimates from 1982 onward. From the censuses, births, and death data, we produced our own intercensal estimates for the 1972 to 1981 period and calculated yearly population exposures. All calculations were made according to the HMD Methods Protocol. The number and boundaries of the provinces of Italy have changed multiple times since 1971, with the number of provinces increasing from 94 to 107 between 1971 and 2016 (Clary & Gargano, 2018). In order to ensure consistency over time of the provincial mortality data, we grouped provinces so that the divisions remain constant from 1971 to 2019. For example, the island of Sardinia experienced three reorganizations of its territory since 1971 (Clary & Gargano, 2018), so we treat the island as a single province in our analysis. This resulted in a total of 90 harmonized provinces. Given that the majority of these 90 units consist of single provinces (or single provinces in the 1970 s that later split), we consider that the results we present are representative of the results we would obtain if we were able to easily extend the current provincial boundaries back in time.

For Spain, we obtained individual death records since 1975 which included information on province of residence, sex, birth cohort, and age at death from the Instituto Nacional de Estadística (INE). We also used intercensal provincial population estimates from 1975 from the INE, and we then calculated yearly population exposures, also following the HMD Methods Protocol. In Spain, no major changes to the boundaries of the provinces have occurred since 1833, so we did not need to harmonize provinces.

For all three countries, some common methods from the HMD protocol were used to make adjustments to the deaths and population data. This included use of the “extinct generations method” and “survivor ratio method” for estimating populations above age 85. The HMD uses these methods above age 80; we opted to use them only above age 85, since these methods assume no migration after this cutoff age. These methods were also already used by Bonnet (2020) for the French HMD. For Italy and Spain, our final datasets had 110+ as the open age interval; however, the data we obtained from the French HMD had 95+ set as the open age interval. To ensure full comparability of results, we set the open age interval at 95+ for Italy and Spain as well. We do not focus on mortality differences at high ages so this will not impact our results.

The final result of our data collection and preparation is three subnational mortality datasets. In our analysis, we considered years 1975 to 2019, since data were available for all countries back to 1975, and we did not want to analyze the effects of the COVID-19 pandemic, which constitutes a rupture in the long-term dynamics we analyze. The principal characteristics of each country’s territorial divisions (as we used in our analysis) are summarized in Table 1.

**Table 1:** Territorial divisions used in the analysis

Country	Territorial division	Number	Min. pop	Max. pop
France	Department	95	73.080	2.604.329
Italy	Grouped provinces	90	83.665	4.358.885
Spain	Province/Autonomous city	52	53.662	6.675.345

After obtaining harmonized series of death counts and exposures for each country by calendar year, age, sex, and province (in the following we may use the term “province” to refer to French departments as well for simplicity), we smoothed the mortality rates across age and time (independently for each province) using *P*-spline methodology (Currie et al., 2004; Camarda, 2008, 2019). Smoothing, in our case, is important in order to distinguish real trends from year-to-year stochastic fluctuations that occur in small subnational populations. Zero exposures in older ages and zero death counts in younger ages are present in our data, and *P*-splines allow us to overcome these issues. Furthermore, smoothing over time is especially justified in our case because we are concerned with long-term trends and not the effects of crisis years (pandemics, heatwaves, etc.) on geographic mortality inequalities. We present selected results without smoothing in the supplementary material to illustrate the necessity of smoothing and serve as a sensitivity analysis.

From the smoothed mortality rates we constructed life tables using standard methods (Preston et al., 2001), and from the life tables we obtained estimates of life expectancy at birth $e^0_{ti}$ for each year *t* and province *i*. We also consider smooth estimated (non-integer) death counts at age *x*, year *t*, and province *i*

$$\begin{aligned} \hat{D}_{xti} = \hat{m}_{xti}E_{xti}, \end{aligned}$$

where $\hat{m}_{xti}$ is the fitted smooth mortality rate for age *x*, year *t*, and province *i*, and $E_{xti}$ is the corresponding observed person-years of exposure. Estimated deaths are used in our analysis of age-specific mortality differences.

### Quantifying Divergence and Convergence in Mortality Between Geographic Areas

To assess subnational convergence or divergence in mortality, we first calculated (for each country) the unweighted standard deviation of life expectancy at birth between provinces for each year:

$$\begin{aligned} \sigma _{t} = \sqrt{\frac{1}{N} \sum _{i=1}^{N}( e^0_{ti} - \overline{e^0_t})^2 }, \end{aligned}$$

where $\overline{e^0_t}$ is the average life expectancy at birth between provinces, and *N* is the number of provinces. The standard deviation of life expectancy provides a straightforward measure of dispersion between provinces and also has the advantage of being measured in the same units of life expectancy. A decreasing value of $\sigma _{t}$ indicates convergence. We opt to study only convergence in $\sigma _t$ and not beta convergence because we complement $\sigma _t$ with other approaches. Essentially, beta convergence reveals if weaker provinces improve faster, catching up with stronger ones. We analyze changes in provincial rankings and life expectancy geography to assess whether they truly caught up or overtook better-performing provinces. We use the unweighted standard deviation because our aim is to examine disparities between places rather than individuals (Bonnet & d’Albis, 2020; Janssen et al., 2016; Hrzic et al., 2023). We show results for the population-weighted standard deviation in the supplementary material, and note that our overall conclusions remain the same.

Multiple scenarios can cause similar changes in $\sigma _{t}$. For example, if $\sigma _{t}$ decreases, this can be due to either worse-performing regions catching up to better-performing regions, with improvements across all provinces (upward convergence), or better-performing provinces experiencing deterioration and approaching worse-performing provinces (downward convergence). In scenarios of increases, decreases, and stability in $\sigma _{t}$, the existing geographic gradient at the start of the observation window can change as well. For example, if worse-performing provinces catch up, overtake, and surpass better-performing provinces $\sigma _{t}$ can eventually increase. To analyze changes over time in the geographic gradients, we first calculated the Kendall rank correlation coefficient (Kendall, 1938) between the ranks of the provinces at the start of the study period (1975) and for each subsequent year until the end (2019). The Kendall correlation coefficient ranges between −1 and 1, with 1 indicating perfect correlation between the ranks of the two sets of observations, and −1 indicating complete discordance between the ranks. A coefficient of 0 means the number of concordant ranks equals the number of discordant ranks. We plotted the trajectory over time of the Kendall correlation coefficient to show how stable the provincial ranking of life expectancies stays.

The Kendall rank correlation coefficient quantifies how much the subnational rankings of life expectancy changed over time, but it does not show us where the changes occurred. Thus, we calculated the difference between each province’s life expectancy and the mean life expectancy:

$$\begin{aligned} \Delta e^0_{ti} = e^0_{ti} - \bar{e}^0_t, \end{aligned}$$

which serves as an indicator of the relative position of each region in the geographic mortality gradient. Positive values mean the province is at an advantage compared to the average, and negative values mean the province is at a disadvantage. We then mapped these differences to identify specific provinces whose relative position of life expectancy changed substantially during the study period.

While life expectancy is useful for collapsing an entire mortality schedule over age into a single indicator, it can be insensitive to changes in mortality in some ages. A change in life expectancy depends on the sum of changes in age-specific mortality, weighted by the proportion of deaths and remaining life expectancy at each age (Vaupel, 1986; Vaupel & Canudas Romo, 2003). In the countries and period we study, overall, very few deaths occur in infancy, childhood, and young adulthood, and life expectancy change is driven mostly by mortality dynamics at older ages. This lack of sensitivity makes the standard deviation of regional life expectancies not a particularly good indicator of mortality convergence at ages where changes in mortality have had little impact on life expectancy. However, inequalities in mortality risk at younger ages are important, since these deaths represent the greatest potential years of life lost.

In order to analyze age-specific mortality inequalities, we calculate for each age and year the index of dissimilarity (Wagstaff et al., 1991; Vierboom et al., 2019) between the distribution of deaths and the distribution of population exposures among the provinces. The index of dissimilarity is defined as:

$$\begin{aligned} ID_{xt} = \frac{1}{2}\sum _{i=1}^{N}\bigg |\frac{\hat{D}_{xti}}{\hat{D}_{xt}} - \frac{E_{xti}}{E_{xt}}\bigg |, \end{aligned}$$

where $\hat{D}_{xti}$ is the number of estimated smooth deaths at single age *x* during single year *t* for province *i*, $\hat{D}_{xt}$ is the total number of estimated smooth deaths in all provinces at age *x* during year *t*, $E_{xti}$ is the person-years of exposure at age *x* during year *t* in province *i*, and $E_{xt}$ is the total person-years of exposure at age *x* and year *t* in all provinces.

The index of dissimilarity is a relative indicator that ranges between 0 and 1. It represents the fraction of deaths that would need to be redistributed between provinces to achieve a scenario where the distribution of deaths matches the distribution of population exposures, representing perfect geographic equality in mortality rates in the given age and year. For example, consider a country composed of only two provinces, A and B, a single age, *x*, and year, *t*. If province A has a population of 90 and 10 deaths, and province B has a population of 60 and 15 deaths, the index of dissimilarity is

$$\begin{aligned} \frac{1}{2} \bigg (\bigg |\frac{10}{25} - \frac{90}{150}\bigg | + \bigg |\frac{15}{25} - \frac{60}{150} \bigg | \bigg ) = 0.20. \end{aligned}$$

The index of dissimilarity is comparable across ages and levels of mortality.

Here, using smoothed deaths is especially important, since the index of dissimilarity is sensitive to zero death counts in very-low-mortality situations. For example, at child ages, if during one year in one province there is a single death and in the others zero deaths, the index of dissimilarity evaluates to 1, indicating maximum inequality, even if the underlying mortality risk is not detectably dissimilar among the provinces. In the supplementary material, we give an example of results using raw data calculated with grouped ages and years to show the value of using smoothed single-year data. As a further robustness check, we show the results obtained by taking the (unweighted) standard deviation of the smooth log-mortality rates in the supplementary material, and we arrive at the same conclusions.

Overall, we opted for what we consider the most easily interpretable indicators, given the goals of our analysis. In the supplementary material, we give examples of alternative indicators of geographic mortality inequalities. We find that our main results are not sensitive to the choice of indicator.

We provide the code used to produce all results in the repository at the following link: https://osf.io/auf58

## Results

### Diverse Trajectories of Subnational Convergence in Life Expectancy

**Fig. 1:**
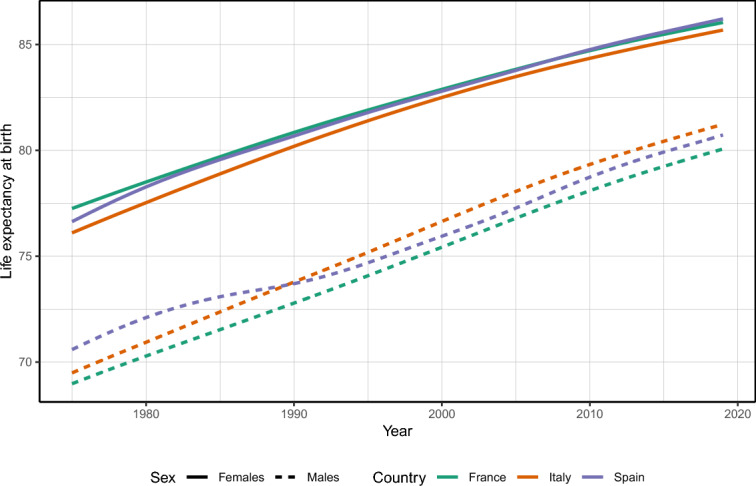
Estimated country life expectancy at birth, France, Spain, and Italy 1975–2019. Overall, sex-specific life expectancy follows similar trajectories and remains at similar levels in the three countries. Source: own calculations done on smoothed mortality rates. Death and population data from French Human Mortality Database, Istituto nazionale di statistica, and Instituto nacional de estadística.

First, to contextualize our results, Figure 1 shows the country life expectancy at birth aggregated to the national level for the three countries. Country rankings shift by sex, but life expectancy starts similarly and rises at a similar pace from 1975 to 2019. Notably, Spanish males’ progress slows around 1985-1995, causing them to lag behind Italian males by 1990.

**Fig. 2:**
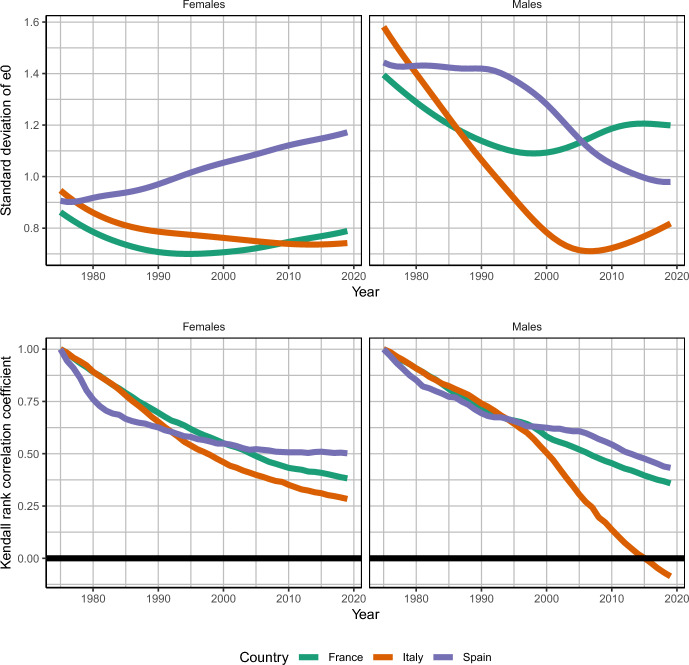
Standard deviation of subnational life expectancy and Kendall correlation coefficient of rankings of subnational life expectancies. **Top:** Standard deviation of life expectancy at birth among subnational units. A higher standard deviation means greater variation in subnational life expectancy, and an increasing/decreasing trend over time indicates divergence/convergence. **Bottom:** Kendall correlation coefficient between ranking of subnational life expectancies in 1975 and subsequent years. A value of 1 indicates perfect correlation between the ranking of life expectancies in a given year and the ranking in 1975. A value of −1 means that the ranking is opposite that of 1975. A decreasing value of the coefficient indicates increasing changes in subnational life expectancy gradient. Source: own calculations done on smoothed mortality rates. Death and population data from French Human Mortality Database, Istituto nazionale di statistica, and Instituto nacional de estadística.

In the top two panels of Figure 2, we show the values for $\sigma _{t}$ for the years 1975 to 2019 for both sexes in all three countries. In the bottom two panels, we show the results for the Kendall correlation coefficient; first, we comment on the results for $\sigma _{t}$. The level of inequality varies greatly between countries, sexes, and over time, with the lowest levels being registered by French females in the mid-1990 s (with a standard deviation of just under 0.7 years). Italian males at the beginning of the period show the highest level of inequality, with a standard deviation of over 1.5 years. For the most part, within each country, we see a greater level of inequality for males than for females, but since the mid-2000 s $\sigma _{t}$ is higher for Spanish females than males, and for Italy for 2002–2011 males show a lower inequality lower level of $\sigma _{t}$ than females. The relative position of countries in terms of the level of $\sigma _{t}$ also changes over time, with Italian males having their highest level of inequality in 1975 but the lowest in 2019.

There is no apparent universal pattern of convergence or divergence: there are cases of both increases and decreases in $\sigma _{t}$, and males and females within the same country may have different trajectories, as is the case for Spain, where females show a sustained increase from 1975 to 2019, and males experience a rapid descent from the mid-1990s to 2019. In France, for both sexes, we observe a pattern of convergence up to the 1990s, followed by divergence until 2019, and for females, the value of $\sigma _{t}$ in 2019 was nearly that of 1975. Italian males also show convergence followed by divergence since the mid-2000s, though inequality between provinces remains much lower in 2019 than in 1975.

Italy and Spain, the two most similar contexts in this study, show a similar pace and level of decrease in regional inequality in life expectancy for males. However, while Italy starts this decrease earlier, Spain begins later but follows a similarly steep negative slope. On the other hand, females in the two countries show trends in opposite directions: while Spanish females experience a constant increase, Italian females have a modest decrease.

Life expectancy at birth increased in every year, country, region, and sex, except for males in the province of Barcelona in Spain between 1983 and 1989. We therefore conclude that the cases of convergence observed (Italian and Spanish males, and both sexes for France between 1975 and 1995) are in fact upward convergence.

Finally, when interpreting $\sigma _t$ in Fig. 2, we should be cautious about pinpointing exact years of trend shifts. Smoothing allows us to discern patterns that would otherwise be obscured by noise, though it does not provide precise change points. While our estimates suggest clear shifts, such as in France and Italian males, the timing may slightly differ. The key takeaway is the sustained increase in $\sigma _t$ since the 1990s. See the supplementary material for raw (unsmoothed) estimates.

### Stability and Reversals of Geographic Gradients

The schematic conclusions on inequality trajectories inferred from the standard deviation of life expectancy in Fig. 2 hide an important dimension: geography. By analyzing the trajectories of the ranking of each province in terms of life expectancy, we assess the stability of geographic gradients in mortality over time. If the relative position of each province’s life expectancy compared to the other provinces in a country remains the same for all provinces, we can conclude that a mortality gradient continues to exist, even if there is an overall upward convergence in life expectancy. On the other hand, in a scenario of upward convergence, there may be a reversal of the gradient if underperforming provinces overtake better-performing provinces. The same is true in situations of divergence, which may be due to a gradient becoming more pronounced or to a reorganization of the relative positions of each province, leading to a new gradient that becomes increasingly pronounced, hence creating divergence.

In the bottom two panels of Fig. 2 we plot the Kendall correlation coefficient between the rankings of provincial life expectancies at birth in 1975 and subsequent years until 2019. The correlation coefficient is 1 in 1975, since this is the start of the series, and will decrease if there is any change in the ranking, which is the case for all of our countries. For females in all countries, there is a similar decrease in the correlation coefficient until the mid-2000 s, after which it stabilizes, indicating some changes in the geographic gradient. Again, for females, the coefficient remains positive in all countries, and not smaller than 0.28, which means that the provincial rankings stay positively correlated to their initial rankings, implying that the overall structure of the geographic mortality gradient is similar.

In contrast, we see a roughly similar pattern for males in France and Spain, but for males in Italy, the correlation coefficient reaches negative values for the most recent years. The negative correlation between the rankings indicates that the life expectancy gradient was partially reversed. In Italy, the reorganization of the provincial rankings led to a situation of lower inequality, since Fig. 2 shows a rapid convergence in provincial life expectancy during the 1975–2005 period. Although the correlation coefficient reaches negative values for Italian males, it is still far from the minimum possible of −1, so not all provinces necessarily saw their ranking change.

**Fig. 3:**
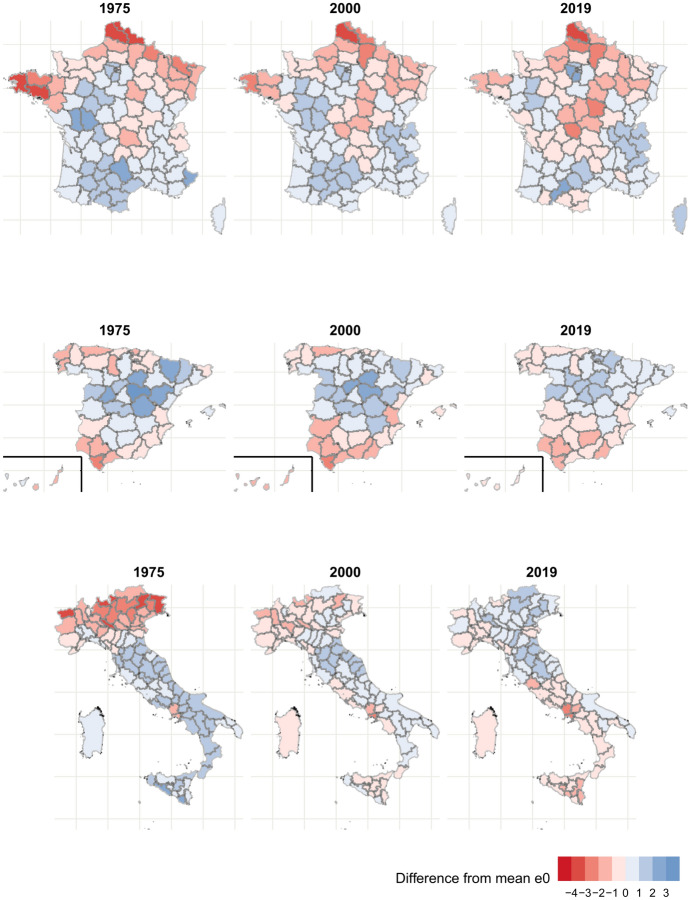
Difference between provincial life expectancy and national average, males. Darker shades of red indicate a larger disadvantage with respect to the national average, and darker shades of blue indicate a larger advantage. Source: own calculations done on smoothed mortality rates. Death and population data from French Human Mortality Database, Istituto nazionale di statistica, and Instituto nacional de estadística.

To identify the structure and evolution of the geography of life expectancy in all countries, we mapped the difference between the national average life expectancy and the regional life expectancy in 1975, 2000, and 2019 for all five countries. In Fig. 3, we show these maps for males. The results for females mirror those of males in France and Spain, but for Italy, the changes over time are more pronounced for males. In each of the figures, a greater negative difference (darker shades of red) means that the region is underperforming in terms of life expectancy with respect to the national average, and a greater positive difference (darker blue) means that the region is outperforming the average. By looking for changes in the map between the beginning and end years, we can characterize how the geographic gradient changed, especially in Italy, where the Kendall correlation coefficient became negative.

In France, the geography of life expectancy remains mostly stable, with consistent disadvantage present in the northernmost departments in the Hauts-de-France region (the northernmost region of France consisting of the departments of Aisne, Nord, Oise, Pas-de-Calais, and Somme) and the westernmost departments in the Brittany region (the northwest coastal region consisting of the departments of Côtes-d’Armor, Finistère, Ille-et-Vilaine, and Morbihan). Furthermore, some departments in the Île-de-France region (the Paris metropolitan area), the South, and the East of the country maintain their performance above the national average. However, the results of the Kendall correlation coefficient indicate that some changes in department performance occurred. The disadvantage of departments in the central diagonal (the “diagonal du vide” or “empty diagonal,” mostly rural areas in the center of the country that have experienced depopulation (Oliveau & Doignon, 2016)) became more pronounced, and the advantage of departments in the southeastern region of Occitania, which was the largest in the country, region declined. The performance of departments in the Île-de-France region further improved, leading it to become the national leader. The French case shows us that within the same country, certain elements of the spatial gradient in mortality can persist and remain quite stable over time (the disadvantage of Hauts-de-France and Brittany), while other parts can change substantially (the decline of the pronounced advantage of some departments in Occitania).

Spanish males also show a largely stable geography of life expectancy. In both 1975 and 2019, the center of the country shows the greatest life expectancy advantage, and the South the worst disadvantage. The Northeast also remains at a disadvantage, though it is reduced by 2019. In both periods, the southern Andalusian province of Cádiz and the two Autonomous cities of Ceuta and Melilla (exclaves bordering Morocco) have the lowest life expectancy, and the central province of Guadalajara ranks among the top three provinces. However, some notable changes occurred between 1975 and 2019. The northern region, including the Basque country, Cantabria, Navarra, and la Rioja passed from disadvantage to advantage. Navarra and the Basque province of Araba ranked in the top 10 in terms of life expectancy in 2019. These northern regions are historically the most industrial. The advantage of the capital province of Madrid also increased substantially, with it ranking second in 2019. Overall, as indicated by the decreasing trend of $\sigma _{t}$, we can see that the magnitude of differences decreased.

In contrast to the French and Spanish cases, Italian males show dramatic changes in the geography of life expectancy, manifested in the reversal of the North–South gradient. For example, the top two provinces in terms of life expectancy in 1975 were Agrigento and Ragusa, both located in Sicily, but they had below- average life expectancy by 2019. In 2019, the northern provinces of Trento and Treviso had the two highest values of life expectancy, but had below average life expectancy in 1975, Trento ranking in the bottom ten provinces. Overall, provinces in the northernmost regions of Valle d’Aosta, Piemonte, Lombardia, Trentino-Alto-Adige, Friuli-Venezia-Giulia, and Veneto had the lowest life expectancies in 1975, but by 2019, many of them had achieved above-average life expectancies. In the South and islands, the reverse is true. However, the advantage of provinces in the central regions of Toscana, Emilia-Romagna, and Le Marche maintained above-average life expectancies throughout the period. The southern provinces of Naples and Caserta in the Campania region had a consistently strong disadvantage during the whole period, and in 2019 registered the two lowest life expectancies.

**Fig. 4:**
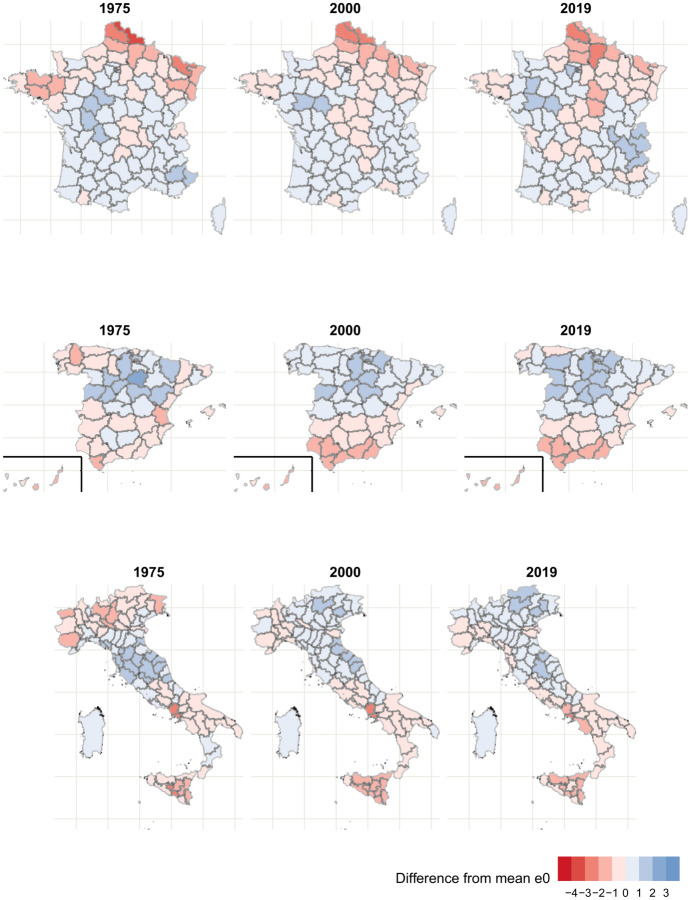
Difference between provincial life expectancy and national average, females. Darker shades of red indicate a larger disadvantage with respect to the national average, and darker shades of blue indicate a larger advantage. Source: own calculations done on smoothed mortality rates. Death and population data from French Human Mortality Database, Istituto nazionale di statistica, and Instituto nacional de estadística.

Figure 4 presents the maps for females. In France, females show results similar to those of males, though the magnitude of differences is smaller. In both 1975 and 2019, the disadvantage of Hauts-de-France and Brittany is smaller for females compared to males, and the same is true for the advantage of Île-de-France. Furthermore, the disadvantage of the central diagonal in 2019 is less clear for females.

In Spain, the overall geographic pattern for females resembles that of males, with a North-Central advantage and a southern disadvantage. However, unlike males, the southern disadvantage becomes more pronounced in 2019, and the performance of the North-Central provinces improves, creating a clearer North–South gradient. The increased differences in life expectancy mirror the results for the standard deviation of life expectancy, since we observed a steady divergence between 1975 and 2019.

In Italy, males and females showed a somewhat different geography of life expectancy in 1975. The provinces in the South of the peninsula and Sicily, which showed a large advantage for males, were at a disadvantage for females in 1975. The northern disadvantage in 1975 is also less pronounced for females. Many northern provinces passed from below- to above-average life expectancy between 1975 and 2019, which helps explain why the Kendall correlation coefficient is the lowest in Italy for females from the 1990 s onwards. In 2019, the maps for males and females are more similar, showing the Center-North outperforming the average and the South lagging behind.

The geographies of life expectancy thus paint very different pictures of convergence. For males, $\sigma _{t}$ decreased between 1975 and 2019 in all three countries. In Spain and France, this corresponded to a decrease in differences between provinces, while the ranking of the provinces remained similar to that of 1975. In Italy, on the other hand, worse-performing provinces in the North caught up to and surpassed initially better-performing provinces in the South, eventually reversing much of the North–South gradient. As the better-performing northern and central provinces left behind the South, a divergence beginning in the mid-2000 s has occurred. For females, a more pronounced north–south gradient accompanied a diverging trend in provincial life expectancy in Spain, and in France, both the geography of and level of inequality in life expectancy were stable. Italian females show less pronounced changes in terms of geography since the southern disadvantage was already present in 1975, but we still see the emergence of the northern advantage.

Despite these quite different trajectories, we observe some similar patterns across countries. In both France and Spain, in the capital provinces of Paris and Madrid, already at an advantage in 1975, life expectancy gains outpaced the average. In Italy, this is not the case, as life expectancy in the Rome province fell below the average between 1975 and 2019, but the opposite is true for the province of the second largest urban area, Milan. In Italy and Spain, the industrial regions of the North (Lombardy in Italy and the Basque Country in Spain) improved their relative position to above average. However, the industrial departments of the North in France remained at a consistent disadvantage.

### Historical and Emerging Divergence at Young-Adult Ages

**Fig. 5:**
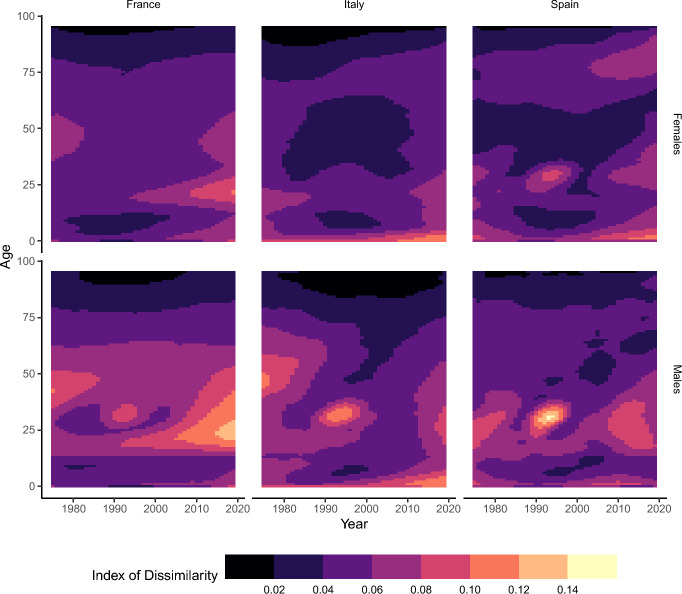
Index of dissimilarity of age-specific mortality. For each age and year, the value of the index is the percentage of deaths that would need to be redistributed in order to equalize the province’s share of deaths with the province’s share of population. Lighter shades on the graph thus indicate greater inequality in age-specific mortality. Source: own calculations done on smoothed mortality rates. Death and population data from French Human Mortality Database, Istituto nazionale di statistica, and Instituto nacional de estadística.

To check whether mortality is converging or diverging at specific ages, Figure 5 shows the values for the index of dissimilarity of mortality between provinces over age and time. For males in all three countries, the mid-1990 s show a peak in the index of dissimilarity for ages between 25 and 35. Although we use all-cause mortality, we know that this is due to the HIV/AIDS epidemic, during which mortality peaked in the mid-90 s in the three countries before the introduction of highly active antiretroviral therapy (Palmisano & Vella, 2011). In all three countries, HIV/AIDS mortality was concentrated heavily in a few urban areas: Paris, Marseille, and Nice for France (Valdes, 2016, 2008); Rome and Milan for Italy (Conti et al., 1992, 1994; Serraino et al., 2004); and Madrid and Barcelona for Spain (Valdes, 2013). Dissimilarity at these ages decreases in the early-mid 2000 s in all three countries, but we see increases in the most recent period. Especially for France, for ages 15 to 30, mortality has started to diverge since the 2000s. This divergence is present but weaker in Italy and Spain.

Male infant and child mortality has diverged as well in France and Italy, with the index of dissimilarity at age 0 increasing since 2000 in both countries and more than doubling in France. In Italy, the index of dissimilarity at ages 1–10 increased sharply since 2000; in France, an increase occurred at these ages, but not as sharply as in Italy. At the national level, infant mortality has increased in France since 2001 (Trinh et al., 2022); our results show that this increase has not been equal across French departments.

At older ages, for males, the diverging trend is less marked, though up to age 50, the index of dissimilarity increased since 2000 in all three countries. The amount of dissimilarity decreases above age 50 in all of the countries, and the trend at each age has remained relatively stable, especially in France.

For females, the magnitude of differences is overall lower than that of males. The HIV/AIDS effect is only visible for Spain, and much less so than for males. The increasing divergence at young-adult ages in France is visible, though again to a lesser degree than for males. The trend in divergence in infant and childhood mortality is also present for females in France and Italy, and childhood divergence is stronger for females than for males in Italy.

At older ages, we see greater dissimilarity for females than for males. At ages above 75 for the three countries, the index of dissimilarity increased; the increase was the greatest in Spain. Overall, taking into account the results for convergence in life expectancy in Figure 2, we can see that life expectancy can show a converging trend, while age-specific mortality follows a diverging trend, as is the case for Spanish males who have shown steady convergence in life expectancy at birth since the late 1990s, but diverging mortality at child, young-adult, and mid-life ages. Italian females also show a small but decreasing trend in $\sigma _{t}$ but diverging mortality at infant, child, and young-adult ages. Furthermore, while for males there is a spike in dissimilarity at young-adult ages in the 1990s for the three countries, it does not translate to an increase in $\sigma _{t}$.

Beyond the impact of HIV/AIDS, age-specific results suggest cause-specific divergences. Since causes of death vary by age, divergences at certain ages imply shifts in those causes. For instance, young-adult divergences may reflect rising external cause mortality. However, as we analyze only all-cause mortality, definitive conclusions on specific causes are not possible.

## Discussion

### Similarities and Contrasts in Neighboring Countries

Our analysis reveals that France, Italy, and Spain exhibit some general similarities in geographic mortality differences at the subnational level. All three countries show similar levels in inequality in life expectancy at birth for both sexes at the beginning of the study period, and we see a similar pattern in convergence in life expectancy for Spanish and Italian males, as well as quite comparable trends for French and Italian females. Furthermore, certain common aspects emerge in the evolution of the geography of life expectancy; namely, the growing advantage of large urban areas (Paris, Milan, and Madrid), alongside the persistence of geographic disadvantages in specific regions and cities (Hauts-de-France and Brittany in France, Andalusia in Spain, and Naples in Italy). When considering mortality by age, clear similarities are evident in the impact of the HIV/AIDS pandemic during the 1990s. Additionally, our analysis reveals an increasing divergence in mortality among individuals aged 15–50 since at least the 2000s, irrespective of whether life expectancy at birth is converging or diverging.

We have also shown that neighboring countries with similar mortality dynamics at the national level can have remarkably different trajectories in terms of geographic mortality inequalities. Due to the differing direction and magnitude of convergence, by the end of the study period, differences between countries in the level of inequality in life expectancy had grown substantially. Even in the case of Italian and Spanish males, which show very similar decreases in $\sigma _t$, the geography of the changes that produced the descent is completely different. Finally, within the same country, different trends emerge by sex. Overall, between-province inequality in life expectancy is lower for females. In Spain, however, males showed a consistent decline in the standard deviation of life expectancy from 1990 and 2019. In contrast, females experienced a steady rise during the whole study period, leading to a higher inter-province standard deviation of life expectancy for females compared to males by 2019. Geographic differences in cohort trends in smoking-related mortality could explain part of the divergence observed for Spanish females, as (Bramajo, 2024) found that lung cancer mortality declined most rapidly for cohorts in Madrid and the Basque Country. Nationally, female smoking prevalence rose sharply from the 1950s to the 1990s, then stagnated and declined by the mid-2000s (Bilal et al., 2014; Rey-Brandariz et al., 2024). While pre-1987 subnational data is lacking, from 1987 to 2020, smoking declined most in the Basque Country and Madrid, while it increased slightly in Andalusia and Castilla-La Mancha (Rey-Brandariz et al., 2024). These regional differences likely explain the divergence in $\sigma _t$ for females observed in our analysis, as regions with lower life expectancy in 1975, such as Andalusia and Castilla-La Mancha, showed less pronounced declines in smoking compared to higher life expectancy regions like Madrid and the Basque provinces.

Smoking is a behavioral risk factor that clearly affects mortality (Fenelon & Preston, 2012), but other social determinants of health could also drive regional variation and trends (Preston et al., 2024). Regionally uneven social and economic conditions, or environmental conditions and their associated trends surely play a significant role. For example, mortality varies in regular ways by education level (Hendi et al., 2021; Hendi, 2024), such that regional variation in educational attainment and expansion thereof could drive changes in geographic variation in mortality. Different paces of educational expansion have been documented in Spain (Manzano et al., 2024) and Italy (Ballarino et al., 2014). Regional patterns in other social determinants of health may drive regional levels and trends in mortality in like manner.

### The Role of Internal Migration

In addition to place-based differences in health conditions, observed spatial inequalities in mortality may be driven in part by selective migration, changing the population composition of different geographic areas. During the study period in all three countries, marked trends in internal migration have been documented, including sustained northern out-migration in France (Baccaïni, 2007), southern out-migration in Italy (Impicciatore & Strozza, 2016), and migration toward the largest urban areas in Spain (García Coll & Stillwell, 1999; Gonzáles-Leonardo et al., 2022). Although the objective of our study is not to determine the exact causes of the observed trends in subnational mortality convergence and divergence, it is plausible that internal migration has played a role in shaping these trends. As noted by (Barbieri 2013, p. 402) in the French context, “those who leave are generally healthier than those who stay, especially in depressed employment areas, and they seldom return to live in their native *départements*.” (cf. Aldea Ramos et al. (2023)). This factor could influence the interpretation of our results, as migration patterns may affect regional mortality dynamics by altering the demographic composition of regions over time. The question is not whether selective migration affects local mortality rates, but to what extent it does, and whether, for a given location, this kind of compositional change dampens or exacerbates other social gradients in health.

Recent research on the United States (Xu et al., 2020; Fletcher et al., 2023) and Spain (Aldea Ramos et al., 2023) that has considered mortality by place of birth in addition to place of residence has found significant differences between inequality measures calculated using the two approaches. However, although place of birth and internal migration are important considerations for interpreting differences in mortality by place of residence, these studies do not conclude that internal migration is the main driver of observed spatial inequalities in mortality. Furthermore, these studies only considered mortality after age 50, and they do not explicitly consider other social determinants of health, nor do we in this paper. More research is needed to determine how much internal migration may drive the level of inequality and the divergence and convergence trends we observe, ideally by accounting for other aspects of social stratification that also impact aggregate mortality.

### Theorizing Mortality Change at the Subnational Level

The quite different scenarios of convergence and divergence that we observe underline the challenge of formulating a comprehensive theory of subnational mortality change. Vallin and Meslé (2005) hypothesize alternating phases of divergence and convergence as mortality improves; however, our study finds no clear pattern of such trends across the countries and sexes examined. For each individual country, the convergence or divergence observed could be part of a longer-term divergence-convergence process that we are unable to fully observe due to the lack of historical data for earlier periods. As Vallin and Meslé (2005) note, an absence of observed divergence between geographic areas may indicate that differences in the determinants of mortality across regions have already diminished significantly. On the other hand, in recent years, mortality has improved at a faster rate in capital provinces or large urban areas (e.g., Paris, Milan, Madrid, Barcelona), which could contribute to the divergence process. Furthermore, divergence is evident among Spanish females, though there are no signs of a subsequent convergence.

Our analysis focuses on all-cause mortality, but divergence-convergence cycles may vary by cause of death. Age-specific trends reveal HIV/AIDS-driven divergence in the 1990s, followed by convergence in the 2000s, a pattern identifiable through prior studies on its geographic spread in France, Italy, and Spain. However, for other causes, we lack the necessary data to determine their role in shaping all-cause trends or to detect cause-specific divergence/convergence patterns that may be obscured at the aggregate level. Vallin and Meslé (2005) suggest that uneven progress in reducing specific causes of death drives divergence, a pattern evident at the national level in life expectancy at birth. To fully test this theory subnationally, cause-specific mortality data are essential.

Our results highlight another issue with theorizing mortality change related to geography. For example, the changing geography we see in Italy presents a particularly challenging case to situate within existing theories of mortality decline. Both Frenk et al. (1991) and Vallin and Meslé (2005) predict that mortality improvements will first benefit the advantaged areas within a country. In Italy in the 1970s, the northern provinces showed the worst performance in terms of life expectancy, and the poorer central and southern regions the best. This creates a challenge in determining which provinces were the most advantaged, as the South has long been economically disadvantaged. Mortality improved more rapidly in the North, leading to convergence with the South. However, in recent years, the North has continued to improve at a faster pace, creating a new divergence. In this sense, the expected order of divergence and convergence appears to be reversed. Thus, we argue for the necessity of a new theoretical paradigm that better explains the complexities in our findings.

### Implications for Mortality Forecasting

Our results challenge the validity of the assumptions often underlying regional mortality forecasts used for subnational population projections. The assumption of long-term convergence, used by the Eurostat projections, may not be appropriate, since we have shown a steady divergence for Spanish females during the whole study period. The results for France also undermine the assumption of convergence: life expectancy diverged between departments for both males and females since the 1990s, leading to a level of inequality in 2019 similar to that of 1975. Furthermore, our analysis of age-specific mortality shows that convergence cannot be assumed for many ages. In all three countries, infant, child, and young-adult mortality have diverged to some extent. The “coherent forecast” (Li & Lee, 2005) approach used in some subnational mortality forecasts (for example those produced by the statistical office of Canada (Statistics Canada, 2022)) assumes a long-term stability in the mortality gradient. This assumption would not be valid for Italian males, given the large changes in the geographic gradient of life expectancy during the study period. The different subnational mortality trajectories across countries and sexes in our study highlights the need for more flexible subnational mortality forecasting methods.

### Limitations

Our results are premised on uniform data quality within countries. If specific regions had lower death registration completeness or systematic issues such as age overstatement, trends in these biases could affect observed trends in convergence and divergence. Similarly, if improvements in death registration happened at different paces in different subnational areas, it could alter the interpretation of regional mortality patterns. It is perfectly plausible, but in our case untestable, that the Italian North–South crossover in life expectancy could be driven by differences in rates of death registration improvement. However, we found no literature on this topic, and we have no evidence to pose this as a hypothesis at this time. As a proxy check for data quality, we computed the Whipple and Myers indices for digit preference for the Italian data and found no indication of major data quality issues or systematic differences between provinces.

Our analysis examines trends in France, Italy, and Spain, but the three different systems of territorial division limit comparability between the three countries. We use NUTS-3 divisions, but the characteristics of NUTS-3 units are not identical between countries. Furthermore, due to the changing territorial division of Italy, we had to coarsen the territorial partition to something that does not exactly correspond to the current NUTS-3 units. In the internal migration literature, several possible methods of cross-national comparison have been proposed (Courgeau, 1973; Bell et al., 2002), which rely on observed empirical regularities between migration intensity and distance. As mentioned in Sect. 2, we do not see the different territorial divisions as a major limitation, since our within-country trends remain valid. In any case, we must be cautious when comparing different systems of territorial aggregation, given the different political and cultural factors that produce those systems. More work is needed to harmonize data between countries to be able to make meaningful demographic comparisons.

Another limitation of this study is the focus on all-cause mortality, which limits insight into the specific causes driving divergence and convergence trends. Cause-specific patterns may be obscured, particularly for less common causes that still impact life expectancy. Moreover, without cause-of-death data, we cannot fully assess how our findings align with theories of mortality change, such as the later stages of the cardiovascular revolution central to Vallin and Meslé (2005). Future research should investigate subnational mortality inequalities by cause over an extended time horizon to assess their contribution to overall trends and identify distinct cause-specific patterns.

## Conclusion

In this article, we quantify the amount of divergence or convergence in subnational mortality in France, Italy, and Spain from 1975 to 2019, revealing a complex and nuanced landscape. While male life expectancy converged in Italy and Spain, France showed a more fragmented pattern. For females, inequality in life expectancy remained relatively stable over the period in France and Italy, but steadily increased in Spain. When considering geography, France and Spain presented consistent life expectancy gradients, whereas Italy saw notably shifts. Our analysis of age-specific mortality inequalities reveals common patterns across countries, especially a recent divergence in mortality at younger ages. Ultimately, our results indicate that no single trajectory of convergence or divergence emerges across these three countries, challenging existing theories and forecasting methods.

## Supplementary Information

Below is the link to the electronic supplementary material.

Supplementary file 1 (pdf 5928 KB)
