# Supplementary material for: Spatial Trends in Mortality Convergence: The Cases of France, Italy, and Spain, 1975–2019
Source: Eur J Popul. 2025 Aug 13;41(1):21. doi: 10.1007/s10680-025-09745-7 (PMC12350882; doi:10.1007/s10680-025-09745-7)
Supplement: Supplementary file 1 — (pdf 5928 KB) [file 10680_2025_9745_MOESM1_ESM.pdf]

Supplementary material to “Spatial trends in mortality convergence: the cases of France, Italy, and Spain, 1975–2019”

1 Grouping of Italian Provinces

Province	Province Group
Agrigento	Agrigento
Alessandria	Alessandria
Ancona	Ancona
Arezzo	Arezzo
Ascoli Piceno	Ascoli Piceno
Fermo	Ascoli Piceno
Asti	Asti
Avellino	Avellino
Foggia	Bari-Foggia
Bari	Bari-Foggia
Barletta-Andria-Trani	Bari-Foggia
Belluno	Belluno
Benevento	Benevento
Como	Bergamo-Como
Bergamo	Bergamo-Como
Lecco	Bergamo-Como
Bologna	Bologna
Bolzano / Bozen	Bolzano / Bozen
Brescia	Brescia
Brindisi	Brindisi
Caltanissetta	Caltanissetta
Campobasso	Campobasso
Caserta	Caserta
Catania	Catania
Catanzaro	Catanzaro
Crotone	Catanzaro
Vibo Valentia	Catanzaro

Chieti	Chieti
Cosenza	Cosenza
Cremona	Cremona
Cuneo	Cuneo
Enna	Enna
Ferrara	Ferrara
Firenze	Firenze
Prato	Firenze
Forlì-Cesena	Forlì-Cesena
Rimini	Forlì-Cesena
Frosinone	Frosinone
Genova	Genova
Gorizia	Gorizia
Grosseto	Grosseto
Imperia	Imperia
Isernia	Isernia
L'Aquila	L'Aquila
La Spezia	La Spezia
Latina	Latina
Lecce	Lecce
Livorno	Livorno
Lucca	Lucca
Macerata	Macerata
Mantova	Mantova
Massa-Carrara	Massa-Carrara
Matera	Matera
Messina	Messina
Milano	Milano
Lodi	Milano
Monza e della Brianza	Milano
Modena	Modena
Napoli	Napoli
Novara	Novara
Verbano-Cusio-Ossola	Novara
Padova	Padova
Palermo	Palermo
Parma	Parma
Pavia	Pavia
Perugia	Perugia
Pesaro e Urbino	Pesaro e Urbino
Pescara	Pescara
Piacenza	Piacenza
Pisa	Pisa
Pistoia	Pistoia
Pordenone	Pordenone

Potenza	Potenza
Ragusa	Ragusa
Ravenna	Ravenna
Reggio Calabria	Reggio Calabria
Reggio nell'Emilia	Reggio nell'Emilia
Rieti	Rieti
Roma	Roma
Rovigo	Rovigo
Salerno	Salerno
Sassari	Sardegna
Nuoro	Sardegna
Cagliari	Sardegna
Oristano	Sardegna
Olbia-Tempio	Sardegna
Ogliastra	Sardegna
Medio Campidano	Sardegna
Carbonia-Iglesias	Sardegna
Sud Sardegna	Sardegna
Savona	Savona
Siena	Siena
Siracusa	Siracusa
Sondrio	Sondrio
Taranto	Taranto
Teramo	Teramo
Terni	Terni
Torino	Torino
Trapani	Trapani
Trento	Trento
Treviso	Treviso
Trieste	Trieste
Udine	Udine
Valle d'Aosta / Vallée d'Aoste	Valle d'Aosta / Vallée d'Aoste
Varese	Varese
Venezia	Venezia
Vercelli	Vercelli
Biella	Vercelli
Verona	Verona
Vicenza	Vicenza
Viterbo	Viterbo

Table 1: Grouping of Italian provinces used in analysis

2 Maps of sub-national territorial units

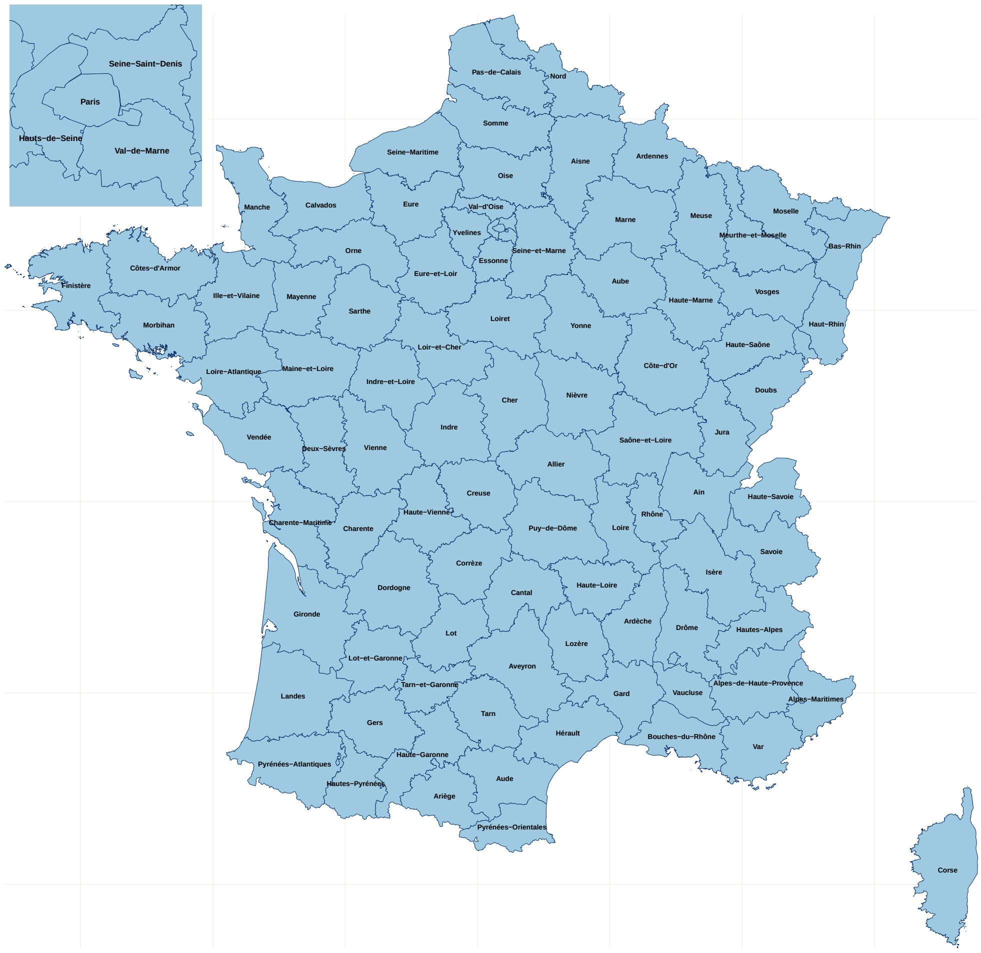

Fig. 1 Map of French departments.

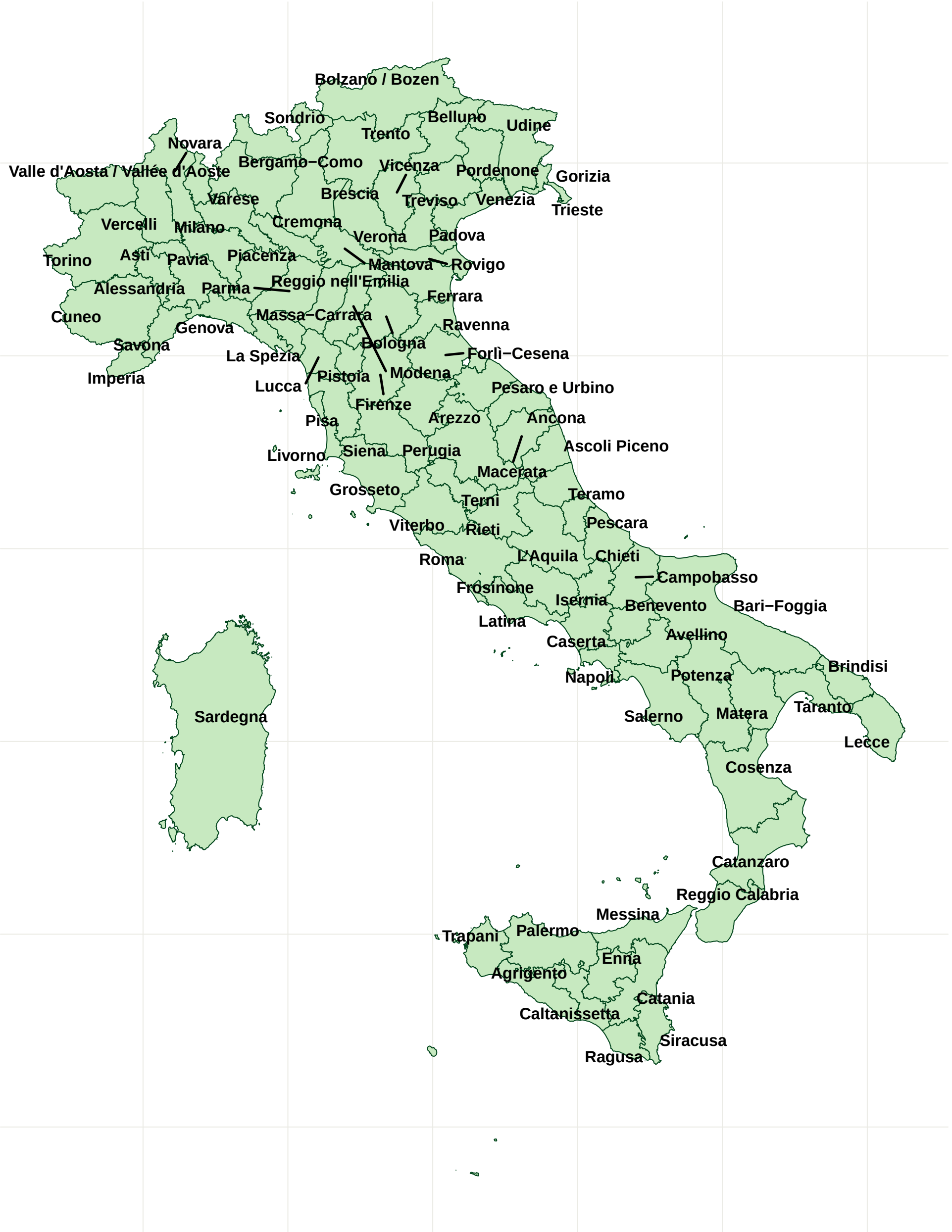

Fig. 2 Map of Italian provinces.

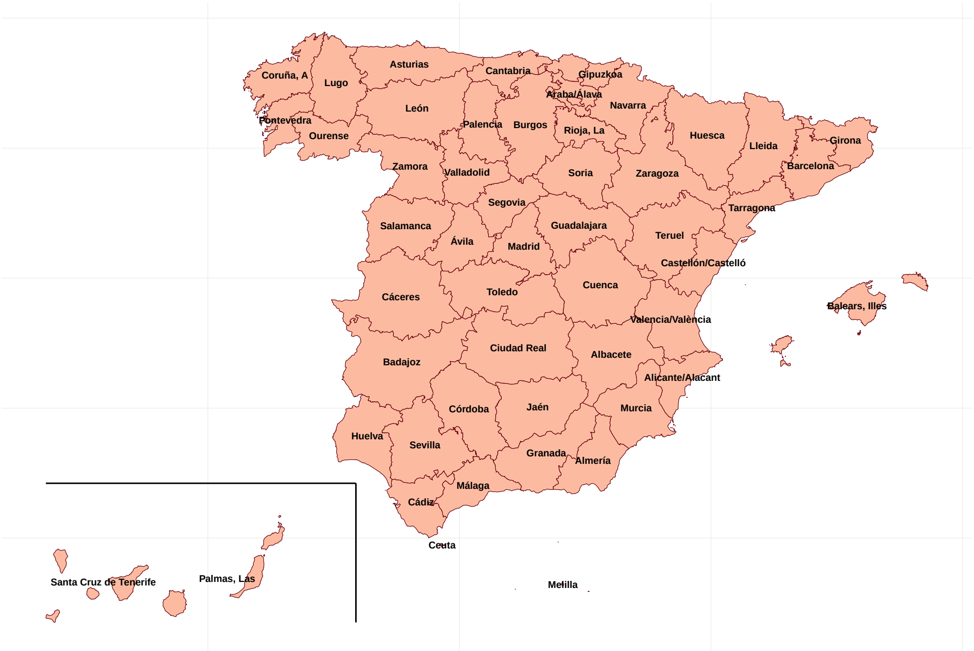

Fig. 3 Map of Spanish provinces.

3 Sensitivity analysis

3.1 Results without smoothing data

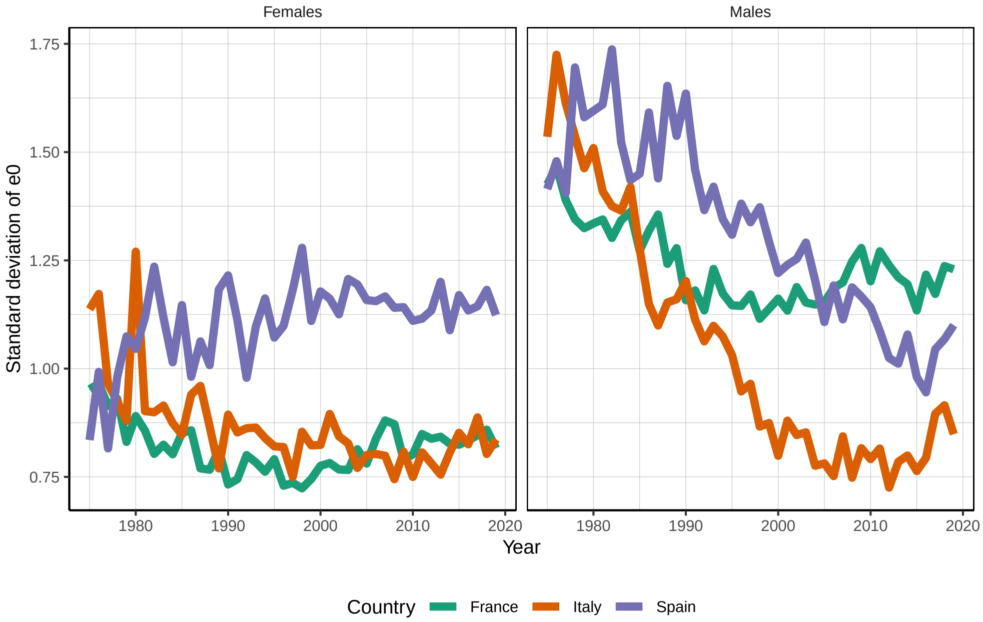

Fig. 4 Standard deviation of life expectancy at birth without smoothing.

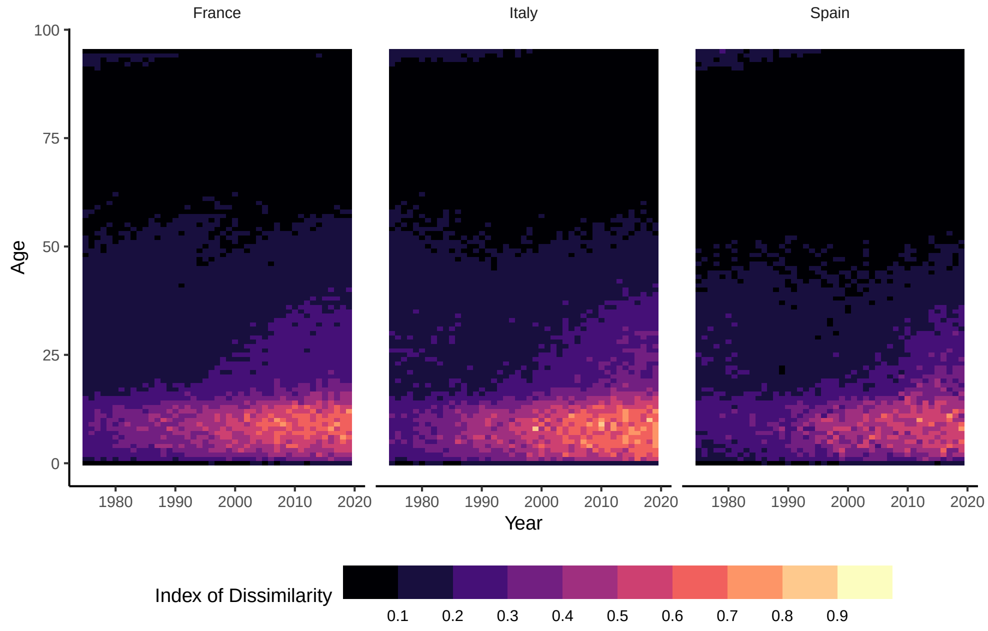

Fig. 5 Index of dissimilarity for males without smoothing (single years of age and time period).

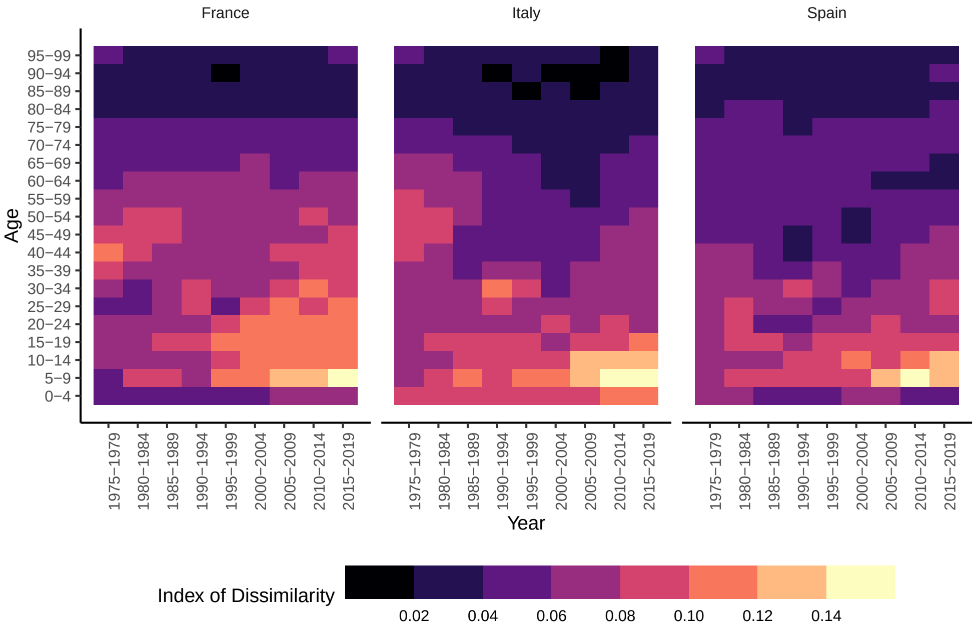

Fig. 6 Index of dissimilarity for males without smoothing (grouped ages and years).

3.2 Results for the weighted standard deviation

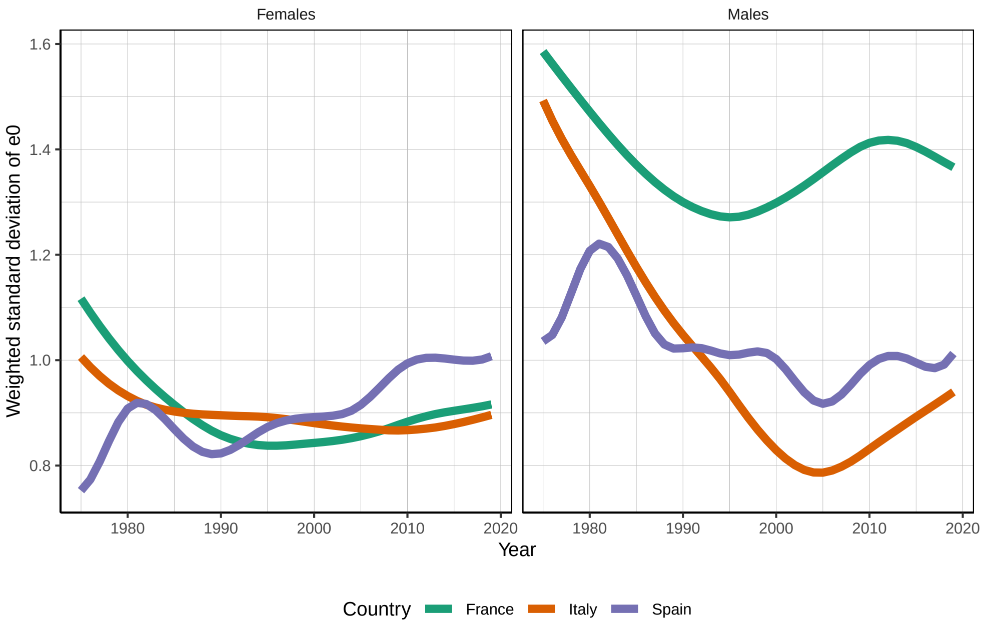

Fig. 7 Weighted standard deviation of life expectancy.

3.3 Relative measures for inequalities in life expectancy at birth

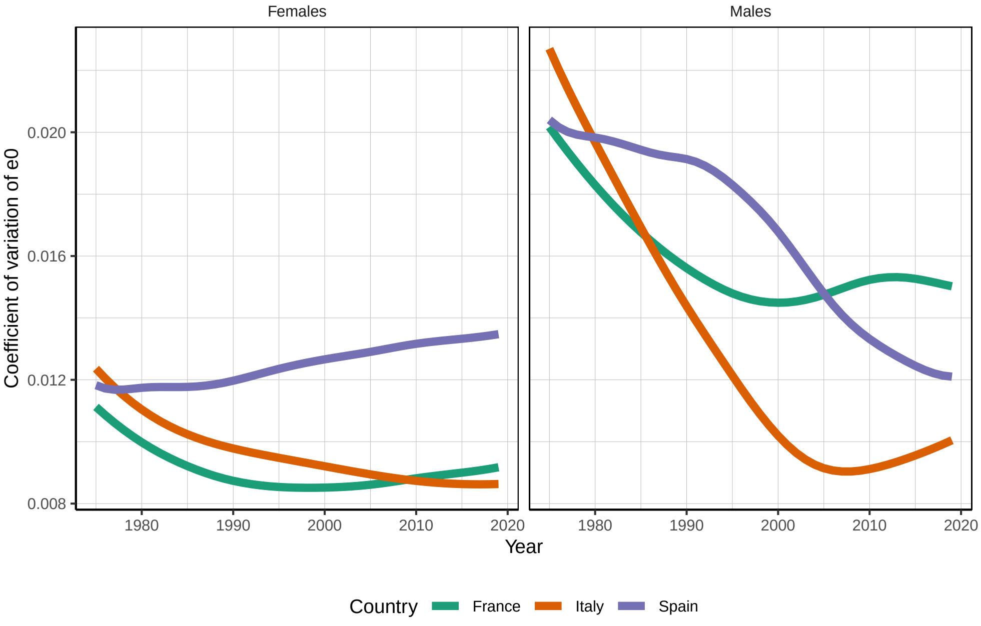

Fig. 8 Coefficient of variation of life expectancy among provinces.

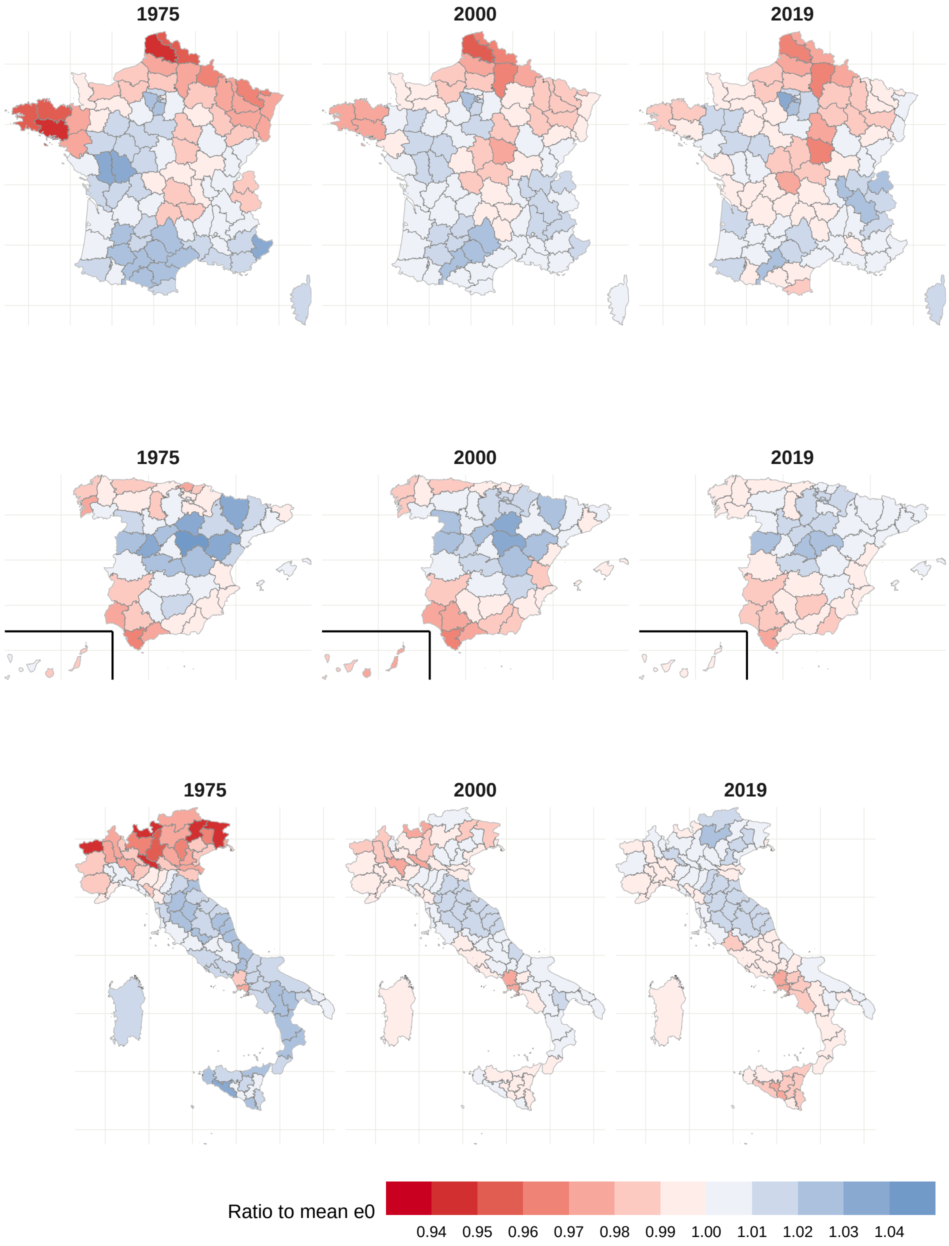

Fig. 9 Ratio between provincial life expectancy and national average, males.

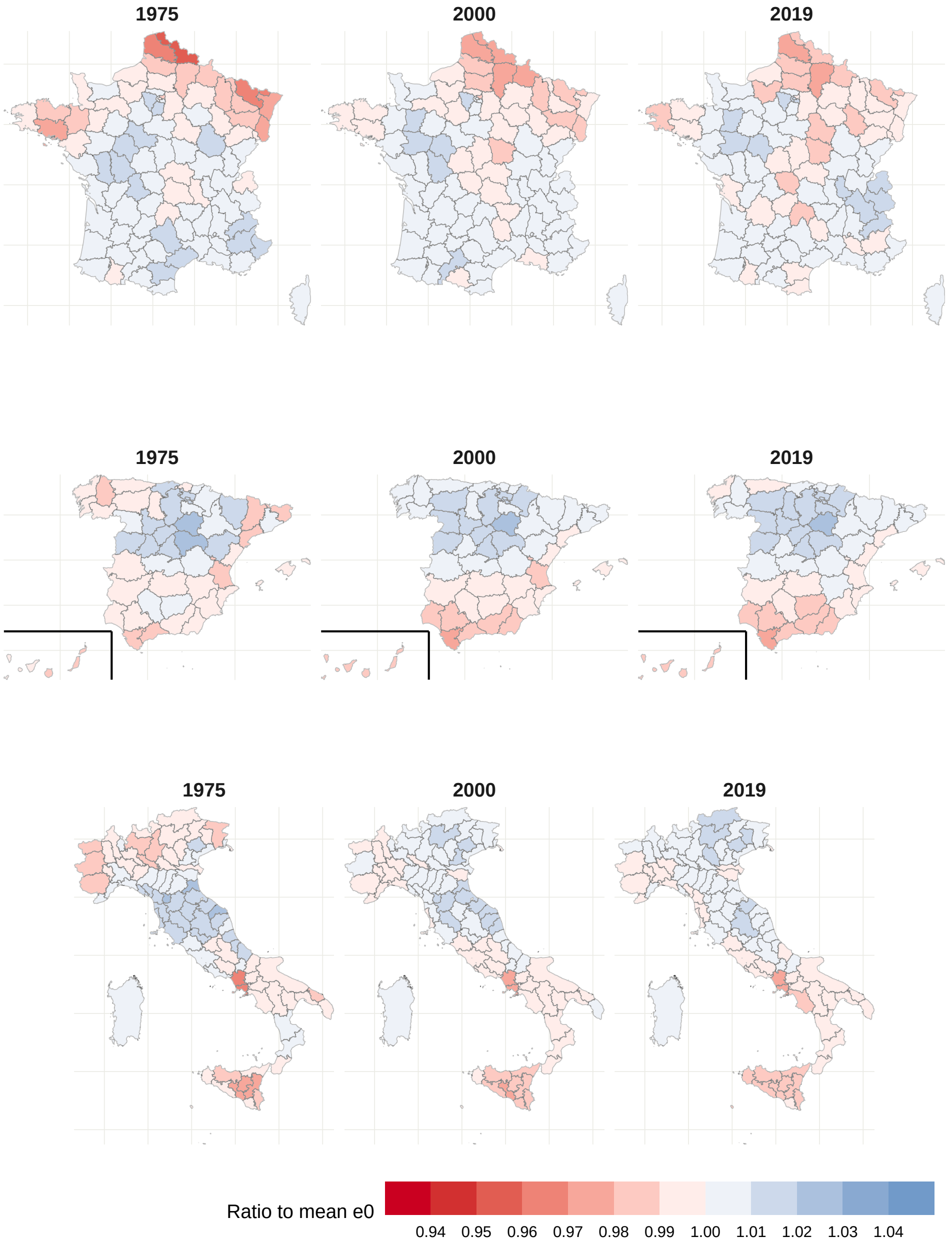

Fig. 10 Ratio between provincial life expectancy and national average, females.

3.4 Alternative indicator for age-specific mortality inequalities

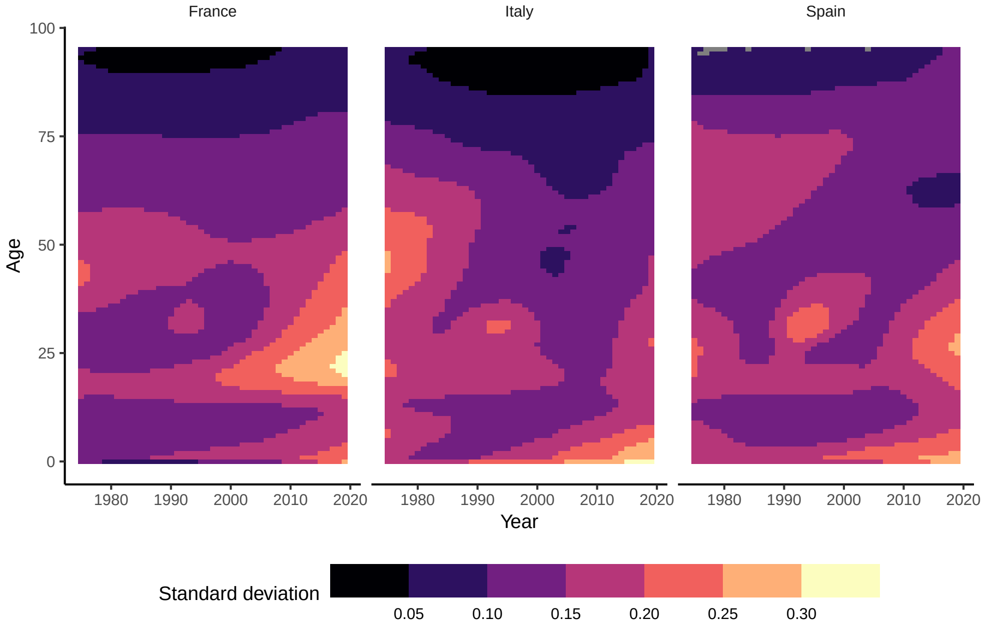

Fig. 11 Standard deviation of log-mortality rates, males.
